# “FAGOMA: Spanish Network of Bacteriophages and Transducer Elements”—V Meeting Report

**DOI:** 10.3390/v10120722

**Published:** 2018-12-18

**Authors:** Modesto Redrejo-Rodríguez, Pilar García

**Affiliations:** 1 Departamento de Bioquímica, Universidad Autónoma de Madrid (UAM) and Instituto de Investigaciones Biomédicas ‘Alberto Sols’ (CSIC-UAM), 28029 Madrid, Spain; modesto.redrejo@uam.es; 2 Instituto de Productos Lácteos de Asturias (IPLA-CSIC). Paseo Río Linares s/n, 33300 Villaviciosa, Asturias, Spain

**Keywords:** bacteriophage, endolysin, phage therapy, biocontrol, genomics, structural biology, evolution, transduction, virulence, antibiotic resistance, biofilms, detection, Spain

## Abstract

The Spanish Network of Bacteriophages and Transducer Elements (FAGOMA) was created to answer the need of Spanish scientists working on phages to exchange knowledge and find synergies. Seven years and five meetings later, the network has become a fruitful forum where groups working on distinct aspects of phage research (structural and molecular biology, diversity, gene transfer and evolution, virus–host interactions, clinical, biotechnological and industrial applications) present their work and find new avenues for collaboration. The network has recently increased its visibility and activity by getting in touch with the French Phage Network (Phages.fr) and with different national and international scientific institutions. Here, we present a summary of the fifth meeting of the FAGOMA network, held in October 2018 in Alcalá de Henares (Madrid), in which the participants shared some of their latest results and discussed future challenges of phage research.

## 1. Introduction: Origin and Composition of FAGOMA

**Figure 1:**
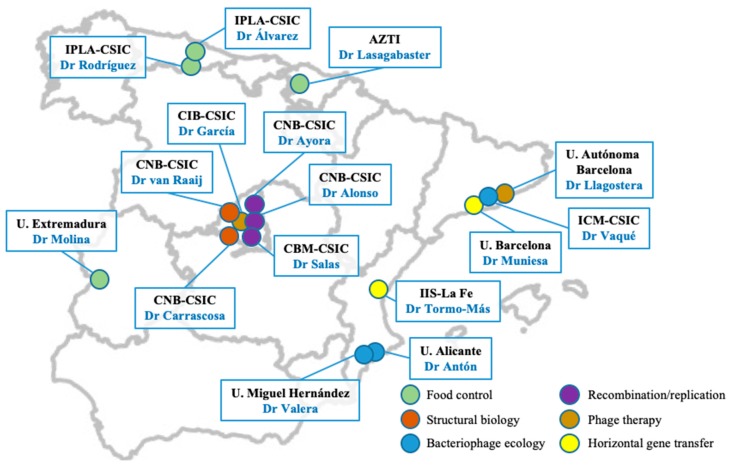
Geographic location of research group members of the Spanish Network of Bacteriophages and Transducer Elements (FAGOMA II) and their main research topic.

As is the case in other European countries [1,2], the Spanish Network of Bacteriophages and Transducer Elements (FAGOMA) was created in 2011 with the aim of bringing together a multidisciplinary group of scientists working in Spain on all aspects of phage biology and applications (Figure 1), as well as encouraging relationships with scientists from related fields. Since that time, our annual meeting has been the perfect setting for promoting and fostering collaborative work between delegates, while also providing a valuable opportunity to discuss their latest results and methodologies.

The network is currently composed of 16 research groups, spanning several universities, technological centers, and institutes from the Spanish National Research Council (CSIC). Moreover, several non-affiliated research groups have also attended our meetings and have expressed their interest in joining the network in future editions (see the Supplementary File for a full list of member groups and attendees of the last meeting). In fact, our last meeting invited delegates from important Spanish research institutions such as Instituto Nacional de Investigación y Tecnología Agraria y Alimentaria-INIA, Instituto de Investigación Biomédica-INIBIC, Instituto de Biología Integrativa de Sistemas-I2SysBio and technological centers such as AINIA.

The best proof of the network’s success is the increase in the number of participants throughout the years, together with the high level of invited speakers. In particular, Dr. Paulo Tavares (CNRS, France), Dr. Francisco J.M. Mojica (University of Alicante, Spain), Dr. Alberto Marina (CSIC, Spain), Dr. Rosa Aznar (University of Valencia and Spanish Type Culture Collection), and Dr. Pascale Boulanger (CNRS, France) have presented inspiring key lectures at our different meetings.

**Figure 2:**
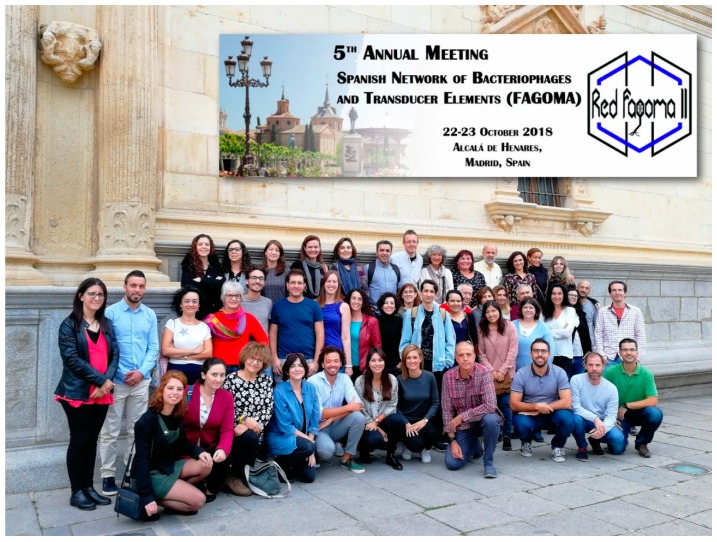
Participants of the V FAGOMA II meeting.

Funded by a grant from the “Redes de Excelencia” networking call from the Spanish government (Ministry of Economy and Competitiveness), FAGOMA was initially set up for the biennium 2011–2012 (Grant Reference BFU2010-10469-E) and resumed as FAGOMA II for the period 2016–2018 (Grant Reference BIO2015-70394-REDT). This funding allowed us to establish a program of short-stay funding and also to hold four successful meetings: two in Oviedo (Asturias) (2012 and 2016) and two in Alcudia (Mallorca) (2012 and 2017). We have recently closed a cycle with a new scientific workshop held last October 22–23 in Alcalá de Henares, near Madrid (Figure 2). The fifth FAGOMA meeting was organized by the network coordinator, Dr. Pilar García from the Dairy Research Institute of Asturias (IPLA-CSIC), and Dr. Modesto Redrejo-Rodríguez, a former member of Professor Margarita Salas’ laboratory at the Center of Molecular Biology Severo Ochoa (CBM-CSIC) and currently at the Madrid Autonomous University.

Inspired by the long history of academic tradition of the University of Alcalá, originally founded in 1499 during the reign of the Catholic Monarchs, 48 phage scientists and aficionados gathered at the “El Bedel” hotel to discuss recent advances in phage biology as well as phage application in biotechnology and as antimicrobials in health services and the food industry.

## 2. FAGOMA II 2018: Summary of Scientific Sessions

In this report, we will outline the oral presentations given at the meeting, which is also a quite precise overview of the topics covered by members of the network. The meeting was organized into five scientific sessions plus two guest speakers that we consider of transversal interest, namely, Dr. Pascale Boulanger (I2BC, Gif-sur-Yvette, France) and Dr. Rosa Aznar (University of Valencia and Spanish Type Culture Collection).

### 2.1. Guest Speaker (I): Dr. Pascale Boulanger

The meeting was inaugurated with an excellent invited talk by Dr. Pascale Boulanger (I2BC, Gif-sur-Yvette, France). Professor Boulanger discussed the work on siphovirus T5 biology that has been carried out in her laboratory throughout the years [3]. Her team has mainly focused on aspects concerning assembly and structure of the viral particle and, more specifically, on the transport of viral DNA into the *Escherichia coli* cell, a process that involves the formation of channels through the cell envelope and subsequent two-step delivery of DNA [4]. She also outlined very recent findings regarding the biological role of the A1 and A2 pre-early genes, which are expressed during the first stage of genome internalization. Although it has been known for 60 years that these genes are essential for resumption of the infection cycle, very little is known about the molecular and cellular mechanisms that they use to control the two-step DNA transfer and host takeover.

Dr. Boulanger is a founding member of the French Phage Network (Phages.fr). For this reason, she dedicated the last part of her talk to introduce in greater detail this network to FAGOMA members. We are hopeful that establishing an ongoing interaction between these two national networks would be the perfect framework to increase future collaborations across the Pyrenees.

### 2.2. Structural and Molecular Biology of Bacteriophages

This session, chaired by Dr. Mark van Raaij (CNB-CSIC, Madrid), began with his PhD student Mateo Seoane, who presented an overview of the crystal structures of several receptor-binding proteins and lytic enzymes from *Escherichia*, *Salmonella*, and *Staphylococcus* phages recently solved by their laboratory [5,6,7], including the structure of the coliphage T5 fibre pb1, a collaboration with Dr. Boulanger’s laboratory. Ongoing structural and binding analysis with receptors and receptor analogs from phages infecting bacteria with an economic or medical interest were also discussed.

Additionally, from the CNB-CSIC, Ester Serrano, from the laboratory of Dr. Juan C. Alonso, gave an inspiring talk entitled “Similarities and differences between natural transformation (chromosomal and plasmid) and viral transfection in competent *Bacillus subtilis* cells”. She addressed the molecular mechanisms by which the RecA mediator (DprA) and modulators (RecX and RecU) regulate RecA filament growth on the internalized single-stranded DNA. These accessory proteins control overlapping mechanisms to process the horizontal transfer of exogenous DNA of different nature, including viral DNA [8,9].

The session was closed by Dr. Annika Gillis, postdoc at Dr. Jacques Mahillon’s laboratory at UCL (Belgium) and recently visiting scientist in the laboratory of Prof. Margarita Salas (CBM-CSIC, Madrid). Since her PhD, Dr. Gillis has been working on the phylogenetic and molecular characterization of tectiviruses, non-enveloped tail-less phages, with a linear dsDNA located within a lipid-containing membrane, covered by a rigid icosahedral protein capsid [10]. She summarized a few newly characterized phages preying on hosts from the *Bacillus cereus* group. The DNA polymerase of some of these phages was at the origin of the collaboration with Prof. Salas’ laboratory, where the DNA replication mechanism of tectivirus Bam35 has been recently characterized [11,12].

### 2.3. Diversity and Evolution of Prokaryotic Viruses

Chaired by Professor Josefa Antón (University of Alicante), this session consisted of only two talks. First, Yaiza M. Castillo from Dr. Dolors Vaqué’s laboratory (ICM-CSIC, Barcelona) presented the premiere of a new, as yet unpublished, method for the detection and monitoring of eukaryotic viruses during their life cycle using multiple specific fluorescent probes. This method allows the study of lineage-specific populations in a more visual and specific manner, giving a better understanding of the potential role played by different virus–host interactions in the biogeochemical cycles in the ocean.

Felipe H. Coutinho, from Dr. Francisco Rodríguez-Valera’s laboratory (Miguel Hernández University in Alicante), presented their recent work analyzing both cellular and viral communities across a depth gradient in the Mediterranean Sea. They retrieved samples at 15, 45 (deep chlorophyll maximum), 60, and 2000 meters and analyzed taxonomic and functional profiles of viruses and hosts as well as genomic variability and mutation rates. The conclusions of this work highlight the diversity among marine phages and how it is affected by host communities and environmental gradients.

### 2.4. Guest Talk (II): Dr. Rosa Aznar

Professor Rosa Aznar (University of Valencia) is the director of the Spanish Type Culture Collection (CECT), the only public Microbial Biological Resource Centre in Spain, which serves as a repository and provider of bacteria, archaea, yeast, and filamentous fungi. She presented the main objectives and quality standards of the collection and also their participation in two novel initiatives for the implementation of high-quality infrastructures for microbial resources. At the international level, the CECT is a founding member of MIRRI (Microbial Resource Research Infrastructure, www.mirri.org), an initiative funded by the European Union (EU), aiming to reduce the current fragmentation of bioresource holdings and information and eliminate duplication and redundancy at the national and pan-European level. The same objectives prompted Professor Aznar and other colleagues to construct MicrobioSpain (www.microbiospain.org), a national network of microbial collections that intend to act as a facilitator for the exchange of information and resources toward the generation of a national node for MIRRI.

Once the advantages of public repositories and databases had been presented, we discussed alternatives for the exchange and sharing of information and resources from bacteriophage collections stored in laboratories belonging to the FAGOMA network members.

### 2.5. Phages as Part of the Bacterial Mobilome

With Dr. Maite Muniesa (University of Barcelona) as a chairwoman, this session started with a great breakthrough. Dr. Nuria Quiles Puchalt, postdoc at Professor José Penades’ laboratory (University of Glasgow, UK), summarized their recent paper [13] disclosing a previously overlooked lateral transduction mechanism by temperate bacteriophages of *Staphylococcus aureus*. She showed that DNA packaging initiates in situ from integrated prophages, and large metameric spans including several hundred kilobases of the chromosome are packaged in phage heads at a very high frequency. Replication before DNA packaging amplified prophage genomes so that lateral-transducing particles are formed during normal phage maturation, transforming parts of the *S. aureus* chromosome into hypermobile regions. By this mechanism, they observed transduction at frequencies 1000-fold higher than previously observed. This mode of transduction is a natural part of the phage life cycle and likely occurs often in diverse bacterial species. Thus, transduction might no longer be considered as an accidental event but rather as another mechanism by which phages have evolved to manipulate their hosts for the sake of their own survival [13,14].

Also concerning *S. aureus* mobile elements, Dr. Angeles Tormo-Más (IISLAFE, Valencia) discussed in her talk the mobilization mechanism of pathogenicity islands (SaPIs) by endogenous phages in clinical strains. She reported a newly identified co-resident prophage in seven clinically-relevant strains. They also investigated the molecular mechanism by which prophages inhibit the master repressor (StI) of the SaPIs [15,16]. Using MW2, a community-acquired methicillin-resistant *S. aureus* strain, they could identify the phage derepressor protein and demonstrated a novel generalized mechanism.

Closing this session, Dr. Lorena Rodríguez-Rubio presented data confirming that horizontal gene transfer (HGT) is not a consequence of an inadequate phage packaging but an important mechanism for the spreading of antibiotic resistance genes. This conclusion was obtained from studying *E. coli* clinical strains carrying antibiotic resistance genes (bla_TEM_, bla_CTX-M-1_-group1, bla_CTX-M-9_-group, bla_OXA-48_, *qnr*A, *qnr*S, *mec*A, *sul1*, and *arm*A). These genes were detected in the phage fraction after induction of bacterial cultures with mitomycin C, and it represents a strategy for dissemination of genes in the environment [17].

### 2.6. Clinical Applications of Bacteriophages

In this session, Roberto Vázquez, from Dr. Pedro García’s laboratory at CIB-CSIC (Madrid), discussed how phage endolysins can be applied against Gram-negative bacteria, thanks to the presence of antimicrobial peptide (AMP)-like elements that destabilize the outer membrane [18]. They have screened a set of *Pseudomonas* infecting phage genomes looking for endolysin genes harboring elements with putative AMP traits, yielding at least one candidate endolysin with bactericidal activity. The potential of this candidate to become a therapeutic agent against Gram-negative infections as well as their non-enzymatic features potentially responsible for all or a fraction of its bactericidal activity are currently under investigation.

Among the invited participants not affiliated with the FAGOMA network, Dr. María M. Tomás (INIBIC-CHUAC, La Coruña), introduced her work on multidrug-resistant (MDR) clinical strains of some of the priority pathogens designated last year by the World Health Organization (WHO) [19,20]. She analyzed the evolution of the Quorum network and the mobilome (plasmids and bacteriophages) in clinical strains of *Acinetobacter baumannii* from the same ICU during a decade, finding that the infective capacity of the bacteriophages isolated in this study was higher against strains isolated at the end of this period of time, which correlates with the presence of a functional Quorum network, whereas original strains carrying a deficient Quorum network were poorly infected [21]. Moreover, she also discussed several ongoing projects on genetic characterization of bacteriophages infecting other MDR clinical isolates from common pathogens in hospital environments, like *Klebsiella* spp. or *Pseudomonas aeruginosa*.

### 2.7. Phage Applications in Biotechnology and Industry

Dr. Victor Ladero from the laboratory of Dr. Miguel A. Álvarez (IPLA-CSIC, Asturias) presented their efforts to isolate new phages infecting *Lactobacillus parabuchneri*, which may constitute biotechnological tools against the accumulation of histamine in cheese and other fermented foods. Histamine is one of the most toxic biogenic amines (BA), according to the European Food Safety Authority (EFSA), and it is highly accumulated in cheese due to the activity of lactic-acid bacteria. They have previously shown that *L. parabuchneri*, a major bacterium responsible for histamine accumulation in cheese, is resistant to pasteurization and is able to produce histamine at refrigeration temperatures. It also adheres to stainless steel surfaces by biofilm formation, which increases its potential to contaminate the cheese during processing [22,23,24,25]. They surveyed a *L. parabuchneri* collection for both lytic and temperate phages. In addition, they examined the available genomes of *L. parabuchneri* looking for complete prophages and viral genes with antimicrobial potential that could also be used as antimicrobial agents against *L. parabuchneri*.

Then, Ibai Nafarrate, who works with Dr. Amaia Lasagabaster (AZTI, Bizkaia), reported new great progress in the isolation and characterization of *Campylobacter* specific phages, a promising non-antibiotic alternative to biocontrol *Campylobacter* spp., the most common cause of reported foodborne-illness in Europe, within the farm-to-fork process. Further characterization, including host range analyses, is ongoing to select the most promising phages and determine their suitability for future development of new campylophage-based products.

The last talk of the session was about an innovative application of bacteriophages as markers for the presence of fecal pollution in contaminated water. Professor Maite Muniesa (University of Barcelona) presented BluePhage [26], a recently released method that allows detection of up to 1 somatic coliphage in under 3.5 h, well within one working day. The method is based on the use of tailored *E. coli* host strains with a replacement of *uidB* and *uidC* genes encoding the transport of the glucuronic acid inside the cell, and an overexpression of *uidA* encoding the enzyme β-glucuronidase. Because of the inability of the strain to incorporate the substrate inside the cell, the substrate only reaches the enzyme after the lysis of the bacterial cells caused by phages. Thus, after phage-mediated lysis, the intracellular accumulated enzyme is released to the medium producing a change of color from yellow to dark blue.

### 2.8. Virus–Host Interactions

Invited for the second time to the FAGOMA meeting, Dr. Pilar Domingo Calap and María C. Cebriá Mendoza (I2SysBio, Valencia), presented their work on virus evolution with mycobacteriophages, in order to obtain high fitness phages to specifically destroy mycobacteria. They employed *Mycobacterium smegmatis* and were able to demonstrate the utility of experimental evolution under controlled conditions to increase phage infectivity and specificity. After serial passages of experimental evolution, they observed virus adaptation to its host. These results may be used as a basis for the study of bacterial resistance and for the development of future therapeutic applications based on bacteriophages.

A combination of phage ecology and state-of-the-art microfluidics technology was then presented by Borja Aldaguer from the laboratory of Professor Josefa Antón (University of Alicante), in collaboration with Dr. Aurelio Hidalgo (UAM). They have set up a digital-droplet multiplex PCR method to identify *Vibrio* strains harboring specific prophages. Positive drops can also be sorted on-chip for further analyses.

The very last talk of the meeting was given by Silvia González from Dr. Pilar García’s laboratory at IPLA-CSIC. They have examined parameters that affect diffusion, propagation, and survival of the *S. aureus* phage phiIPLA-RODI in bacterial biofilms. Their results confirmed that the phage could penetrate through biofilms formed by several bacterial strains and it differed depending on the specific bacterium or combination of bacteria. They suggest that factors determining the diffusion rate of phages in biofilms include the amount of attached biomass, the susceptibility of the strain, initial phage titer, phage entrapment in the extracellular matrix, and phage inactivation [27,28]. This work will help to further characterize phage–bacteria interactions within biofilm communities and will be valuable for the development of anti-staphylococcal products based on these phages.

## 3. Perspectives

Progress in any field of research is necessarily accompanied by a multidisciplinary approach and phages are no exception. In our network meetings, there is a confluence of scientists with diverse expertise working on topics that range from basic to applied research. Thus, in this meeting, we realized that spotlight subjects could be tackled from different perspectives. A clear example is the antibiotic resistance threat, which can be approached by developing biocontrol products based on phages and phage-derived proteins or by studying phage-related mechanisms involved in resistance gene spreading. Another example is the role of viruses in natural ecosystems, which is being studied using novel detection systems and metagenomics analysis. Indeed, the full potential of phage applications in diverse fields including clinical, veterinary, and food safety sciences, would require basic research in phage biology, structure, and interaction with the host that will allow us to develop new tools to fight the most threatening pathogens.

Although satisfied with the previous development of the network, we still have great challenges ahead. First and foremost, we have agreed to apply for the next funding call, so that we can continue working with a renovated network (FAGOMA III) that would be expanded with new members. We also aim to continue with our short-stay program, which in the future may support not only the mobility of researchers within the network but also international collaborations. Indeed, we hope that our contact with the French Phage Network could be the beginning of a larger European network. A renovated website including an English version is also on our to-do list.

With all of this in mind, we face a new stage of the FAGOMA network with renewed strength arising from the gratifying scientific sessions shared during this meeting.

## Supplementary Materials

The following are available online at http://www.mdpi.com/1999-4915/10/12/722/s1, Table S1: Full list of member groups and attendees of the last meeting.
