# Supplementary material for: “FAGOMA: Spanish Network of Bacteriophages and Transducer Elements”—V Meeting Report
Source: Viruses. 2018 Dec 18;10(12):722. doi: 10.3390/v10120722 (PMC6316384; doi:10.3390/v10120722)
Supplement: Supplementary file 1 [file viruses-10-00722-s001.pdf]

Table S1: Full list of member groups and attendees to the last meeting

Guest speakers

Dr Pascale Boulanger Institute for Integrative Biology of the Cell (I2BC), CEA, CNRS, Université Paris-Sud & Université Paris-Saclay, 91190 Gif-sur-Yvette CEDEX, France.
pascale.boulanger@i2bc.paris-saclay.fr

Dr Rosa Aznar Departamento de Microbiología y Ecología, Colección Española de Cultivos Tipo (CECT); Universitat de València, Av. Dr. Moliner, 50, 46100, Burjassot, Valencia, Spain.
rosa.aznar@uv.es

Network members

Centro Nacional de Biotecnología, CSIC. Madrid

J. Alonso lab:

Dr Juan C. Alonso jcalonso@cnb.csic.es

Dr Ester Serrano eserrano@cnb.csic.es

S. Ayora lab:

Dr Silvia Ayora Hirsch sayora@cnb.csic.es

Dr Laura Torralba ltorralba@cnb.csic.es

M. van Raaij lab:

Dr Mark van Raaij mjvanraaij@cnb.csic.es

Mateo Seoane mseoane@cnb.csic.es

Centro de Biología Molecular "Severo Ochoa", UAM-CSIC. Madrid

M. Salas lab:

Dr Modesto Redrejo Rodríguez modesto.redrejo@uam.es

Present Address: Biochemistry Department (UAM)

Dr Annika Gillis annika.gillis@gmail.com

Centro de Investigaciones Biológicas, CIB - CSIC. Madrid

P. García lab:

Dr Pedro García pgarcia@cib.csic.es

Roberto Vázquez rvazquez@cib.csic.es

Instituto de Productos Lácteos de Asturias. IPLA-CSIC, Villaviciosa

M.A. Álvarez lab:

Dr Victor Ladero ladero@ipla.csic.es

Dr Beatriz del Río Lagar beadelrio@ipla.csic.es

A. Rodríguez lab:

Dr Pilar García Suárez pgarcia@ipla.csic.es

Dr Ana Rodríguez anarguez@ipla.csic.es

Dr Susana Escobedo s.escobedo@ipla.csic.es

Dr Lucía Fernández lucia.fernandez@ipla.csic.es

Dr Beatriz Martínez bmf1@ipla.csic.es

Silvia González silvia.gm@ipla.csic.es

Eva González eva.gm@ipla.csic.es

University of Glasgow, UK

Penades lab:

Dr Nuria Quiles Puchalt Nuria.Quiles@glasgow.ac.uk

Instituto de Ciencias del Mar, ICM-CSIC Barcelona

D. Vaqué lab:

Dr Dolors Vaqué dolors@icm.csic.es

Yaiza M. Castillo yaiza@icm.csic.es

Universidad Autónoma de Barcelona - UAB

M. Llagostera lab:

Dr Montserrat Llagostera montserrat.llagostera@uab.cat

Dr Mª. Pilar Cortés mariapilar.cortes@uab.cat

Universidad de Barcelona – UB

M. Muniesa lab:

Dr Maite Muniesa mmuniesa@ub.edu

Dr Lorena Rodríguez Rubio lorenarodriguez@ub.edu

Instituto de Investigación Sanitaria La Fe IISLAFE. Valencia

Mª Angeles Tormo lab:

Dr Mª. Ángeles Del Tormo Más tormo_man@iislafe.es

Patricia Bernabé patricia_bernabe@iislafe.es

Universidad Miguel Hernández – UMH. Alicante

F. Rodríguez Valera lab:

Dr Francisco Rodríguez Valera frvalera@umh.es

Rafael González Serrano rafael.gonzalezs@umh.es

Felipe Hernandes Coutinho fhernandes@umh.es

Universidad de Alicante - UA

J. Antón lab:

Dr Josefa Antón anton@ua.es

Borja Aldaguer borja.aldeguer@ua.es

Fernando Santos fernando.santos@ua.es

AZTI-Tecnalia. Euskadi

AZTI lab:

Dr Amaia Lasagabaster alasa@azti.es

Ibai Nafarrate inafarrate@azti.es

Universidad de Extremadura

F. Molina lab:

Dr Felipe Molina fmolina@unex.es

Other participants

AINIA, technological center

Dr Alex Martínez amartinez@ainia.es

Dr Ana Torrejón atorrejon@ainia.es

I2SysBio & University of Valencia - UV

Dr Pilar Domingo Calap Pilar.Domingo@uv.es

María C. Cebriá Mendoza maconce@alumni.uv.es

Complejo Hospitalario Universitario A Coruña-INIBIC (SERGAS) - A Coruña

Dr María M. Tomás Carmona MA.del.Mar.Tomas.Carmona@sergas.es

INIA - Madrid

Dr Sonia Garde sgarde@inia.es

Dr Marta Ávila arribas@inia.es

Dr Javier Calzada jcalzada@inia.es

Dr María C. Sánchez López
